# Use of intraoperative ultrasound in differentiating adamantinomatous versus papillary craniopharyngiomas and guiding resection through the endoscopic endonasal route

**DOI:** 10.1007/s00701-025-06449-z

**Published:** 2025-02-12

**Authors:** Chandrima Biswas, Moataz D. Abouammo, Ludovica Pasquini, Guilherme Mansur, Mohammad Bilal Alsavaf, Kyle C. Wu, Ricardo L. Carrau, Daniel M. Prevedello

**Affiliations:** 1 Department of Neurosurgery, The Ohio State University and Wexner Medical Centre, Columbus, OH USA; 2 Department of Otorhinolaryngology, The Ohio State University and Wexner Medical Centre, Columbus, OH USA

**Keywords:** Craniopharyngioma endonasal endoscopic intraoperative ultrasound

## Abstract

Craniopharyngiomas (CPs) are surgically challenging tumors. The prevalence of BRAF mutation in papillary craniopharyngioma (PCP) and the positive response with BRAF-MEK inhibitors have shifted the treatment paradigm towards targeted therapy. However, maximal safe resection remains the goal, particularly for adamantinomatous craniopharyngioma (ACP). In this report, we describe two cases of CP where intraoperative ultrasonography (IOUS) was helpful in differentiating the subtype of CP, thus enabling intraoperative decision-making regarding the extent of resection. Additionally, IOUS images of three more (two PCP and one ACP) patients who underwent endoscopic endonasal resection for CPs were retrospectively evaluated. Each of these entities showed characteristic appearances on IOUS.

## Introduction

Craniopharyngiomas (CP) are histologically benign and rare tumors that typically arise in the suprasellar region. They lie in close proximity to the optic chiasm, hypothalamus, and arteries of the circle of Willis, making complete surgical resection a challenge and potential for morbidity. As per the WHO 2021 classification for CNS tumors [10], they comprise two separate entities: papillary (PCP) and adamantinomatous craniopharyngiomas (ACP).

The recent identification of a targetable mutation (BRAF V600E) in over 90% of PCPs [3] and the efficacy of the BRAF/MEK inhibitors [4] have shifted the focus of the management of these tumors from radical resection to targeted therapy after pathological confirmation. However, efforts to identify effective targeted interventions for ACPs are ongoing, and surgical resection remains the mainstay of management [8, 2].

Although the surgical goal has remained maximum safe resection, a less aggressive surgical strategy may be considered in select cases of PCP.

Intraoperative ultrasound (IOUS) is an invaluable tool in neurosurgery and can aid in the identification of the pathological subtype and guide the extent of resection. We report two cases of endoscopic endonasal resection of CP where IOUS helped to identify the type of CP and thus guide intraoperative decision-making. The required informed consent was taken from each patient and the study was approved by the Institutional Review Board.

## Case illustrations

### Case 1

**Fig. 1:**
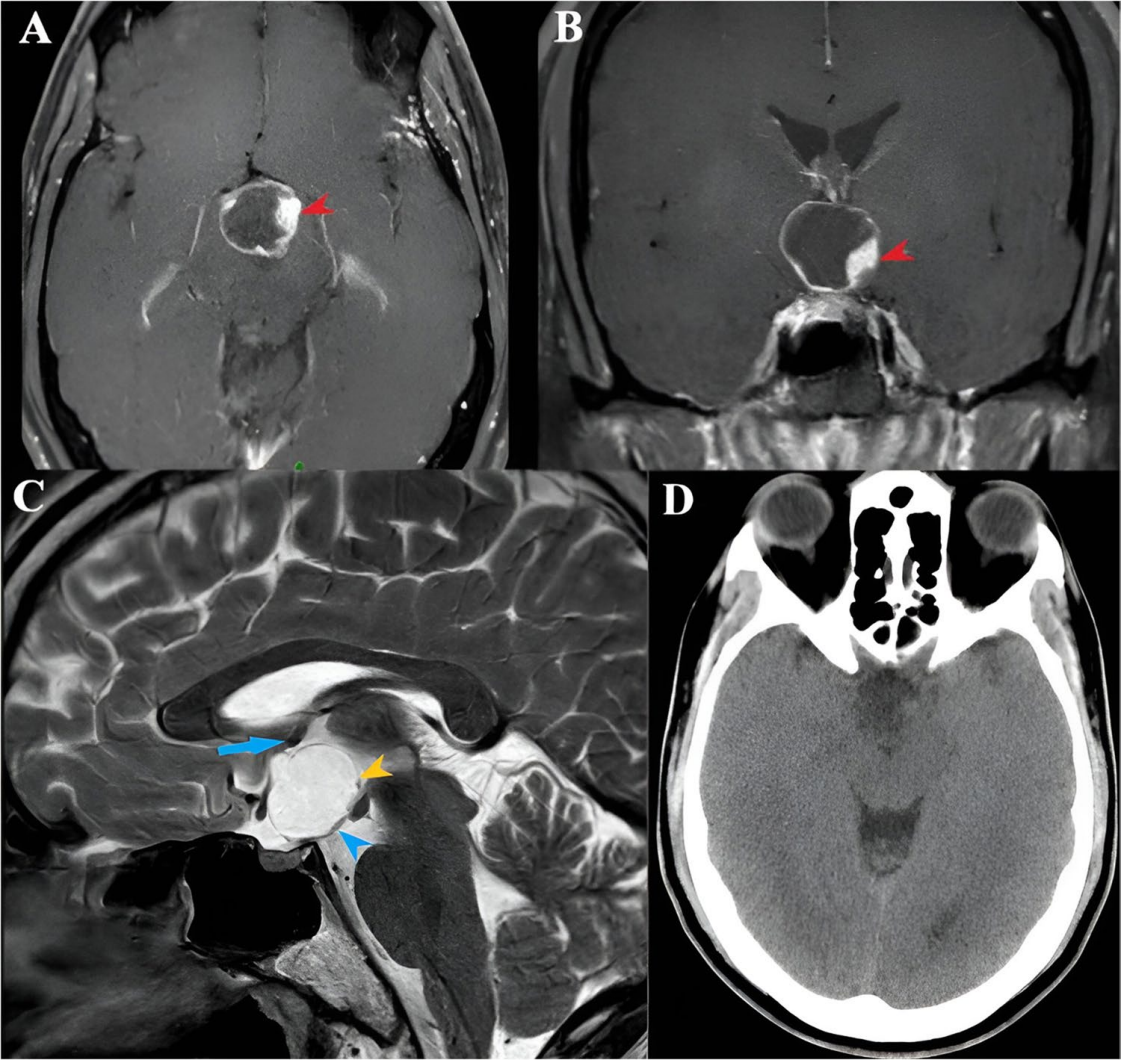
Preoperative imaging of case 1: **A**. and **B**. - Axial and coronal T1 with contrast MRI images showing the contrast enhancing nodule (red arrowhead) and cyst with irregular enhancing wall, **C**.- sagittal MRI images showing a cystic lesion in the suprasellar space pushing the optic chiasm (blue arrow) anteriorly and pituitary stalk (blue arrowhead) posteriorly and floor of the third ventricle (yellow arrowhead) superiorly, **D**. CT image show no calcifications

**Fig. 2:**
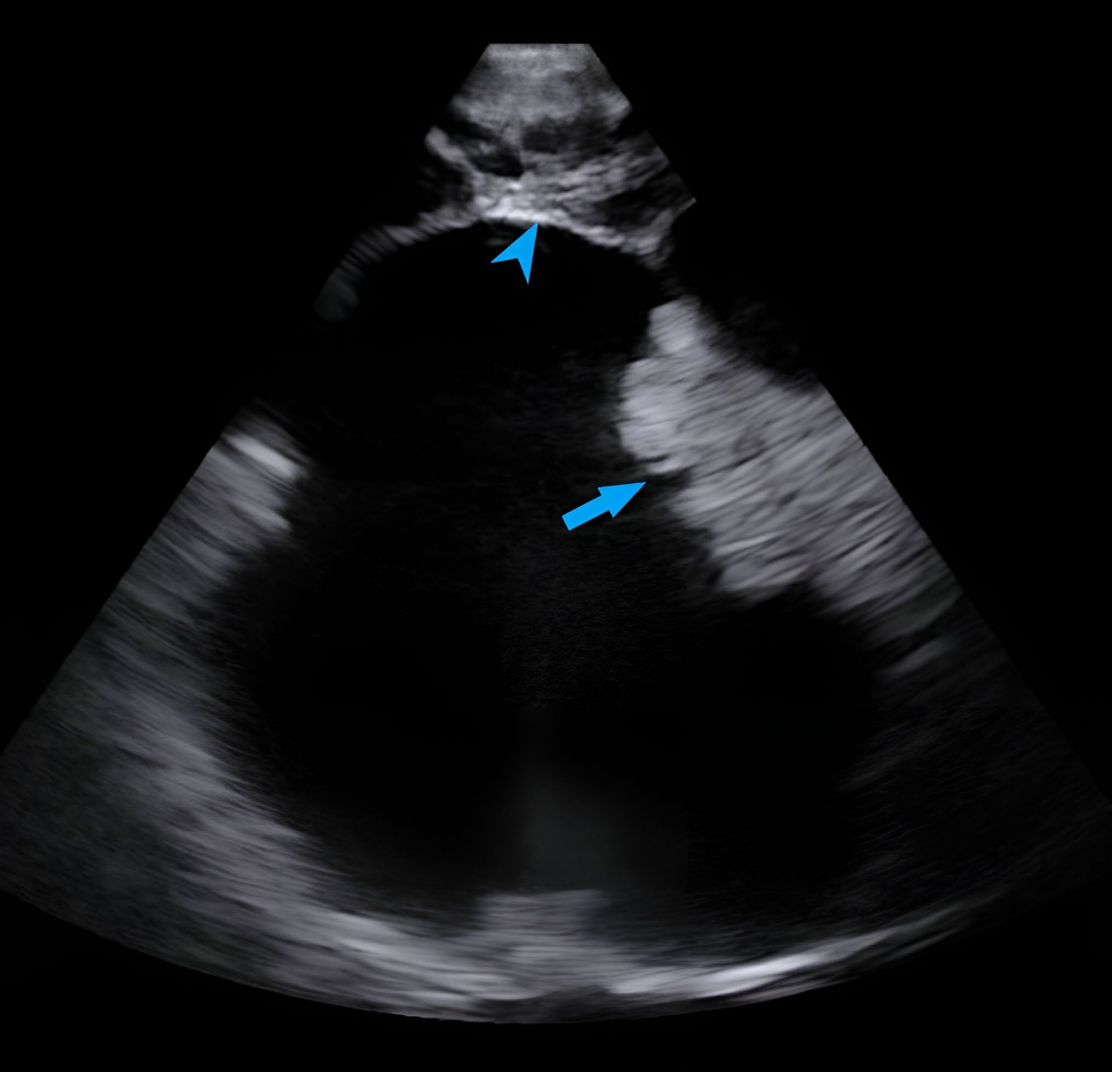
Intraoperative ultrasound images of Case1 (taken after achieving hemostasis and before incising the dura) showing the homogenously hyperechoic solid component with lobulated borders (blue arrow) surrounded by hypoechoic cyst with distinct margins (blue arrowhead)

**Fig. 3:**
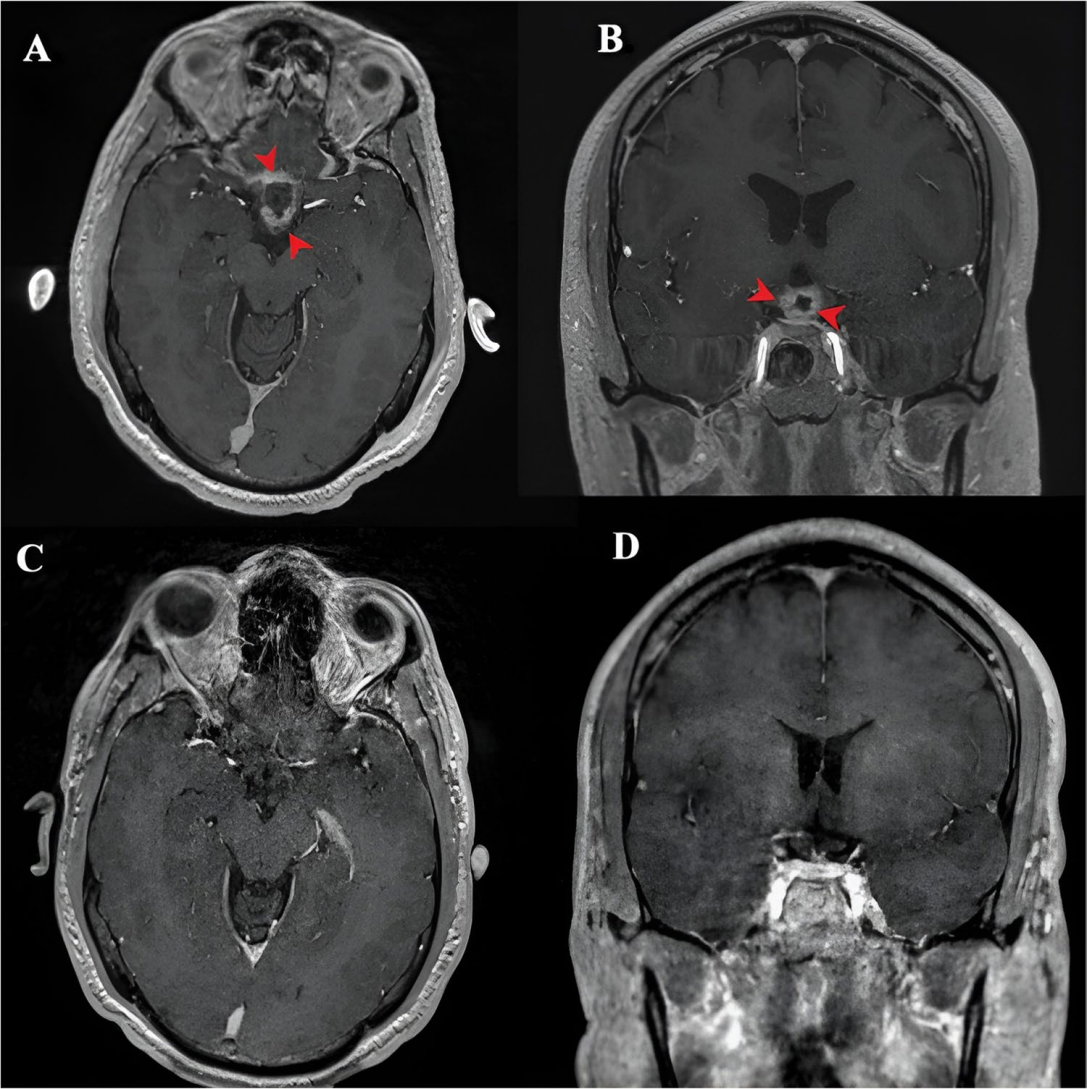
**A**. and **B**.- Immediate postoperative axial and coronal T1 with contrast MRI images of Case 1 showing residue (red arrowhead), **C**. and **D**.- Axial and coronal T1 contrast MRI images after completion of 19 months of dabrafenib + trametinib therapy showing complete resolution

 A 43-year-old gentleman presented with bilateral visual blurring, headaches, and weight gain. On evaluation, he was found to have bilateral visual field defects with mild inferior temporal field deficits. A brain magnetic resonance imaging (MRI) (Fig. 1) showed a large cystic lesion in the suprasellar location with a small enhancing nodular component antero-superiorly; on computed tomography (CT) scan (Fig. 1d) there were no calcifications. The preoperative impression was of a suprasellar craniopharyngioma, possibly PCP. He underwent IOUS-guided resection of the lesion via an endoscopic endonasal trans-tubercular approach. The steps of the procedure have been described elsewhere [15, 13]. After drilling the bone of the sella and the tuberculum sellae, a minimally invasive USG probe (bk5000; BK Medical, 6 × 7 mm footprint and 15 cm shaft length, 20-6 MHz, N20P6 minimally invasive transducer) was introduced into the field, and IOUS images and video were acquired in the coronal plane. It showed a hypoechoic cyst with a well-demarcated hyperechoic wall. The central nodule appeared as a homogenously hyperechoic mass with lobulated margins, giving it a ‘frond-like appearance (Fig. 2). No hyperechoic regions, suggestive of calcification, were noted. The cyst was decompressed, and most of the nodular component and the posterior cyst wall were resected. The intraoperative frozen section reported likely craniopharyngioma but was not specific of which type. A portion of the solid component adherent to the pituitary stalk and optic chiasm was left behind based on the IOUS findings that helped us to lean towards the papillary subtype. Postoperatively, the patient had no neurological or hormonal issues, and his vision improved. The histopathology was suggestive of a BRAF V600E mutant PCP, and the patient was started on dabrafenib-trametinib treatment for the residual disease along the pituitary stalk (Fig. 3a and b). There was a significant reduction in the size of residual disease one month after starting the therapy (Fig. 3c and d) and the lesion has remained stable in the last follow-up − 19 months since the initiation of therapy.

## Case 2

**Fig. 4:**
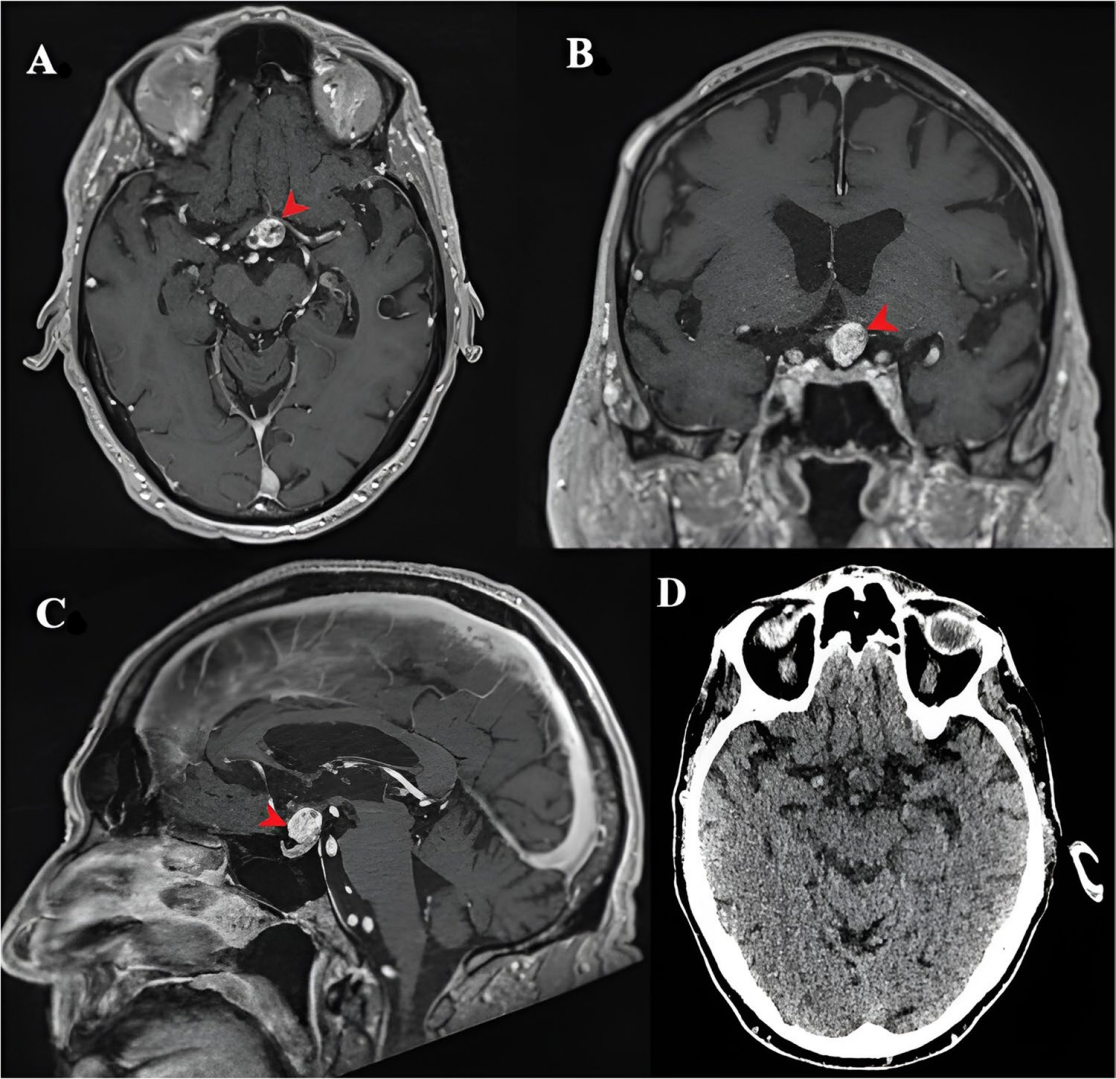
Preoperative imaging of Case 2: A to C.- Axial, coronal and sagittal T1 with contrast MRI images showing a solid enhancing lesion (red arrowhead) in the suprasellar space, D. CT image shows no calcification

**Fig. 5:**
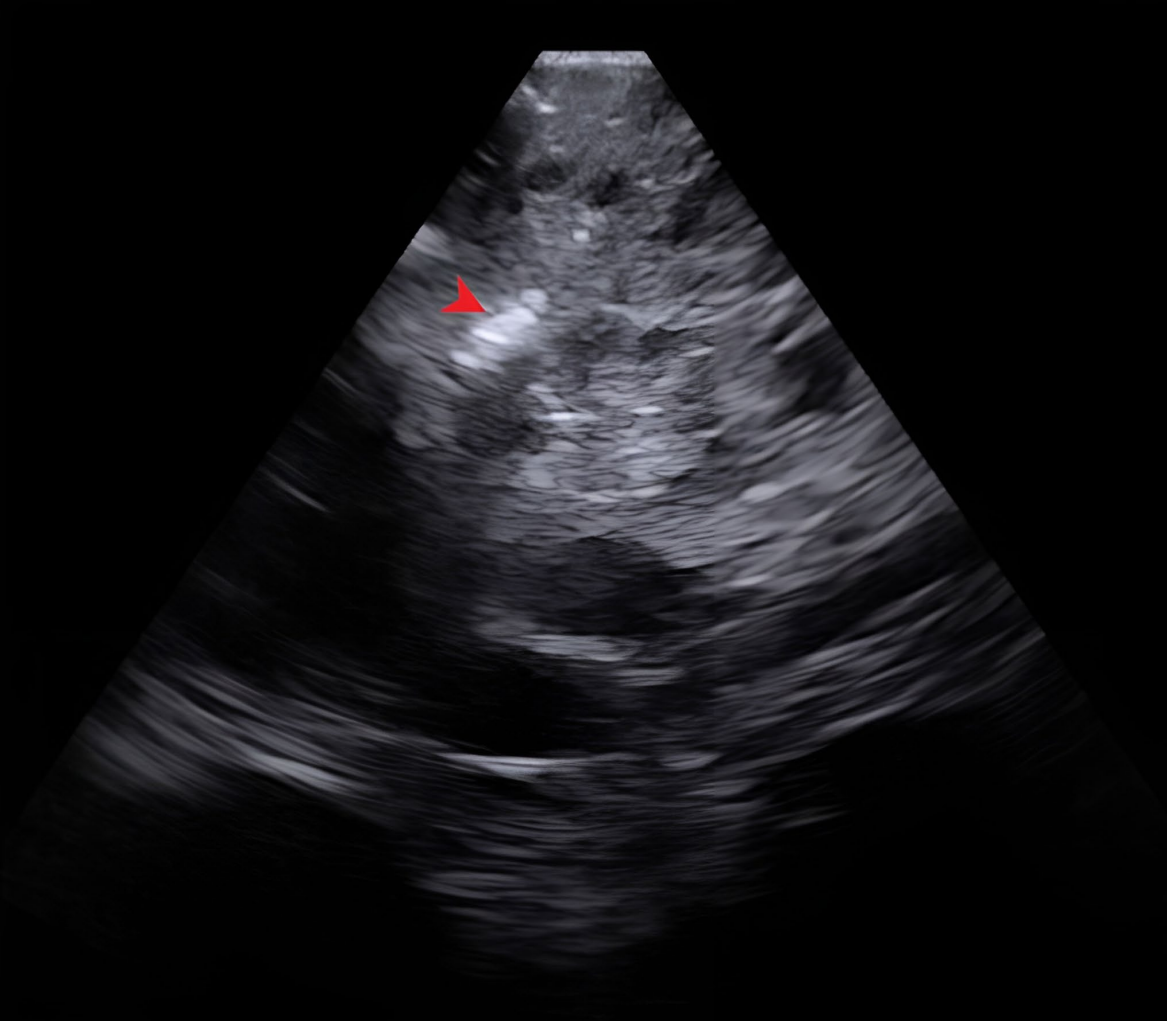
Intraoperative ultrasound images of Case 2 (before incising the dura) showing a heterogeneous lesion with few very hyperechoic areas suggestive of calcific foci (red arrowhead) along with the acoustic shadow. Hypoechoic cystic areas are also seen

**Fig. 6:**
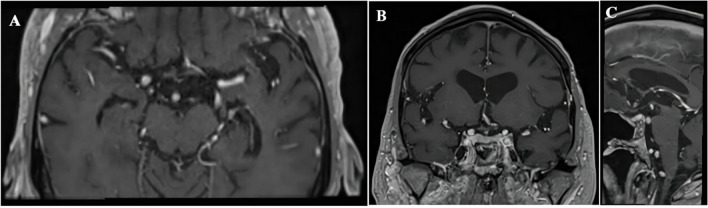
Postoperative T1 weighted MRI with contrast done 6 weeks post resection, shows a gross total resection of the tumor with preservation of the pituitary stalk and optic chiasma

A 48-year-old gentleman presented with a 5-month history of visual blurring. An ophthalmological examination showed right temporal hemianopia and left inferior quadrant field defect. A brain MRI showed a predominantly solid lesion in the suprasellar space involving the pituitary stalk and compressing the optic chiasm (Fig. 4). There was no calcification noted on the CT scan (Fig. 4d). A preoperative impression of PCP was made, and the patient underwent endoscopic endonasal trans-tubercular resection of the tumor. After removing the bone of the sella and tuberculum sellae, a durotomy was performed, and an USG probe was introduced. It showed a heterogeneously hyperechoic lesion which was not very well demarcated from the pituitary gland. There were several hyperechoic areas with acoustic shadows suggestive of calcific foci (Fig. 5). This was suggestive of ACP. Dural exposure was widened, and the diaphragma sellae was cut and the tumor was identified in the suprasellar space. Given the suspicion for an ACP, gross total resection was pursued with preservation of the optic apparatus and the pituitary stalk. Histopathology confirmed an ACP, and the postoperative MRI scan showed no residue (Fig. 6). The patient had subjective improvement in vision with no endocrinological dysfunction.

**Fig. 7:**
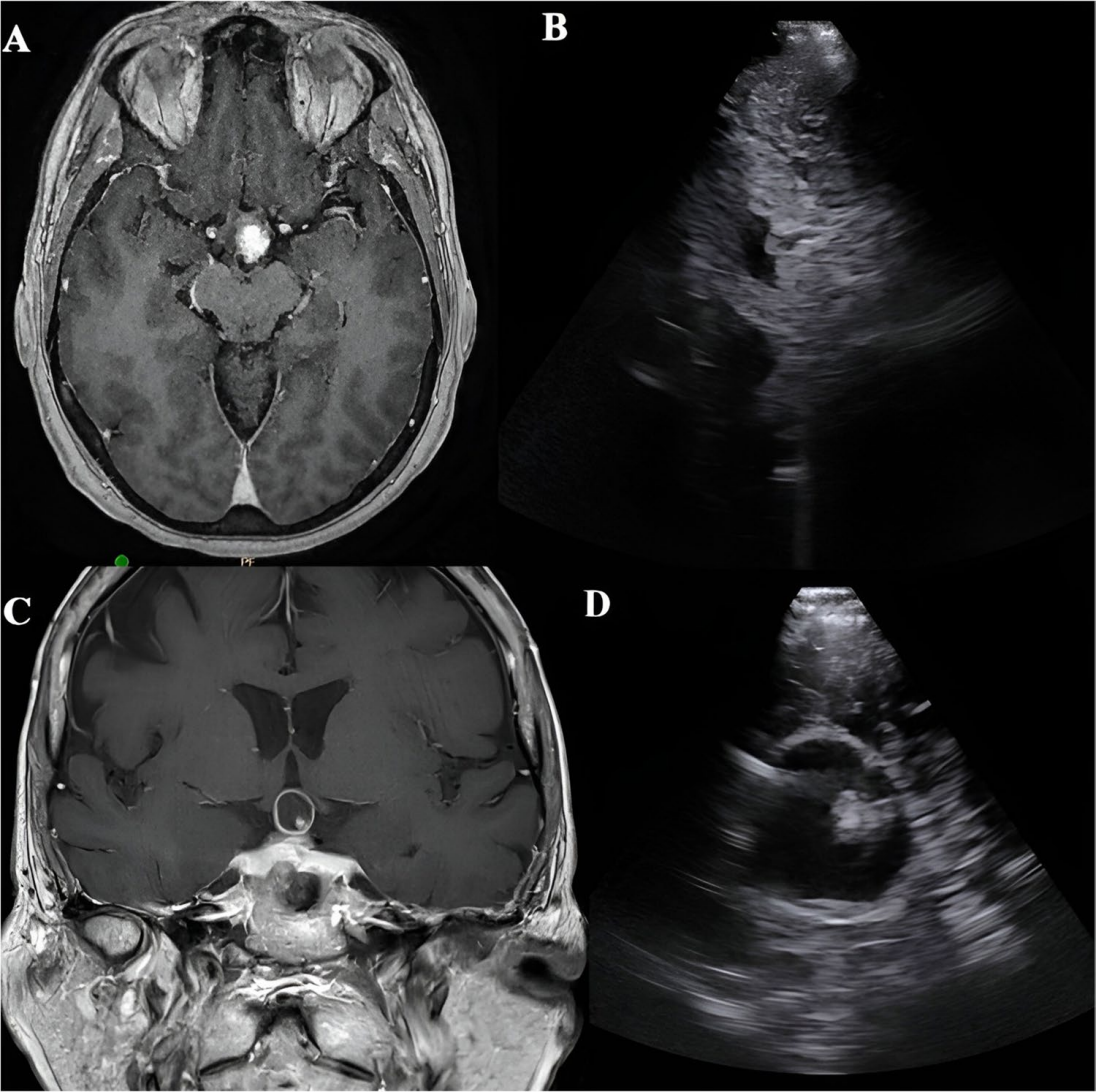
Preoperative MRI images of 2 other cases of PCP with corresponding intraoperative IOUS image (all taken before the dural incision): A. and B.- Preoperative axial T1 with contrast MRI image and the corresponding intraoperative IOUS image of a case of PCP (third patient in the series), C. and D.- Preoperative coronal T1 with contrast MRI image and the corresponding intraoperative IOUS image of another case of PCP (fourth patient in the series)

**Fig. 8:**
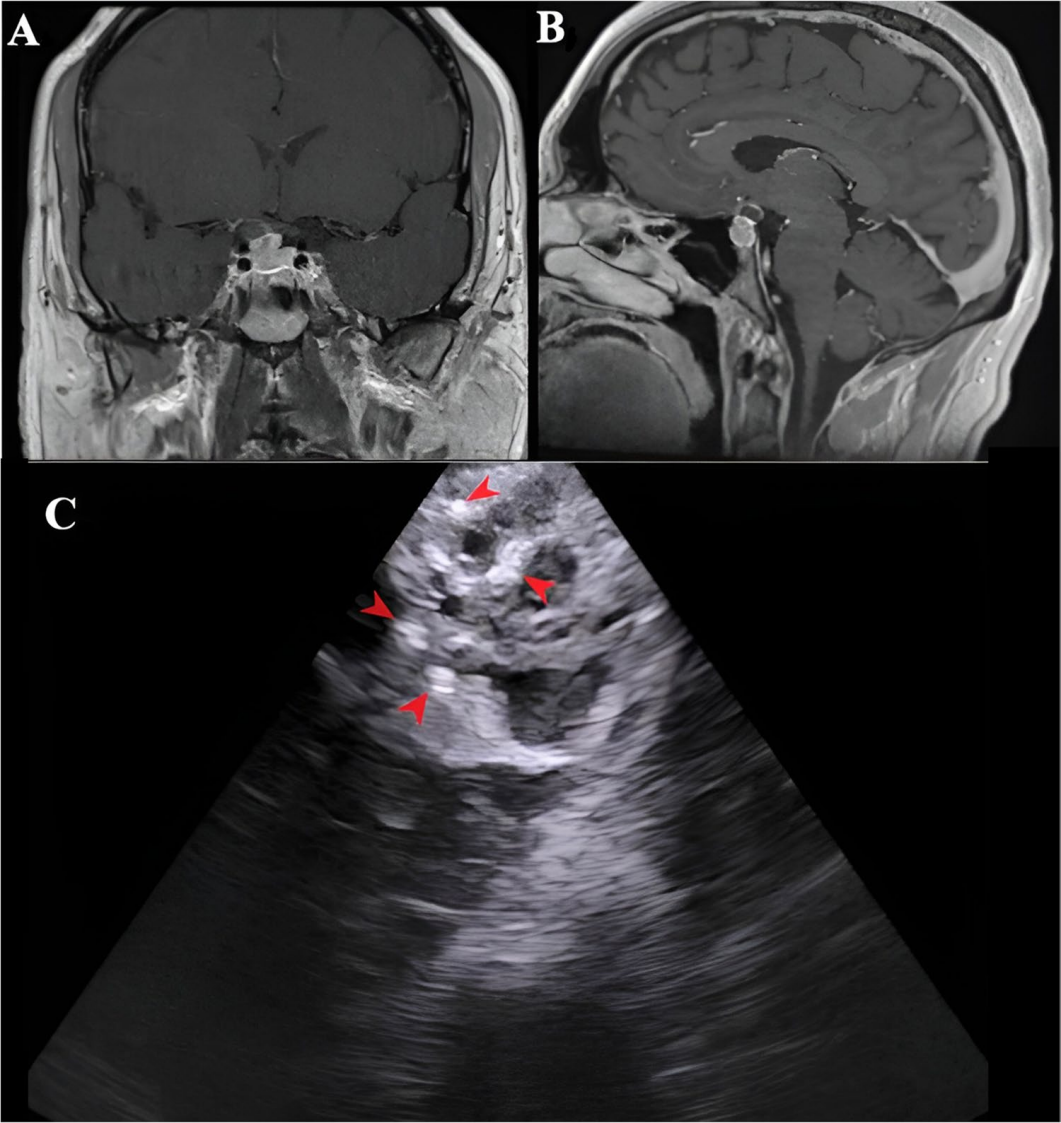
(**A**) and (**B**) Preoperative axial and coronal T1 with contrast MRI images of another case of ACP (fifth patient in the series), (**C**) Corresponding IOUS image showing the typical features with calcific foci (red arrowheads) This figure is original to this submission, so no credit or license is needed

To further investigate the characteristic IOUS features of CPs, IOUS images of five consecutive (three PCP and two ACP) patients who underwent endoscopic endonasal transsphenoidal resection for CPs were retrospectively evaluated (Figs. 7 and 8). The following IOUS features were studied: echogenicity, internal heterogeneity, margins, cysts, and calcific shadows.

PCPs showed solid components with homogenous hyperechogenicity with lobulated margins. They were surrounded by a larger cyst with a well demarcated hyperechoic wall.

ACPs on the other hand had poorly demarcated borders with higher internal heterogenic echotexture with hyperechoic calcific foci and acoustic shadows, giving it a ‘salt and pepper’ appearance [6].

## Discussion

Intraoperative ultrasonography is a valuable, multipurpose, and cost-effective tool used for tumor localization and resection control. It can demonstrate pathologic characteristics of tumors such as hemorrhage, cysts, and calcifications within the lesion as they alter the echotexture [12, 1]. It is even better than MR in diagnosing some tumors intraoperatively [14]. The functionality of this device in the confined space of endoscopic surgery has been addressed by the introduction of smaller footprint transducers, which have undergone several iterations of refinement over the years [11]. In this report we have shown the utility of IOUS in identifying characteristic features of each type of CP and thus helping in intraoperative decision-making regarding the extent of resection.

Identification of specific driver mutations in craniopharyngiomas, particularly in PCP, has shifted the treatment paradigm towards targeted therapy [3, 4]. However, these changes have not been reflected in the management of ACP, and surgical resection continues to be the mainstay of treatment. In the absence of preoperative diagnostic tests that can determine with certainty the specific tumor type, surgical intervention for pathological confirmation is paramount. However, although intraoperative frozen section, and squash and smear cytology can detect CP, it can be difficult to discern the variant [7]. Hence, alternative preoperative or intraoperative methods are required to guide surgical treatment.

On MRI, over 50% of the craniopharyngiomas have a pathognomonic appearance. They are intrasellar and/or suprasellar masses. The solid portions appear isointense or hypointense on T1-weighted MRI (T1W) and hyperintense on T2-weighted MRI (T2W) images but can also have a mottled appearance owing to calcifications. Cystic components are hyperintense on T1W images owing to their high protein or hemorrhagic content [7]. Tumor calcification is appreciated on CT scan and is seen in 83% of ACP [9] and is never found in PCP. Thus, although calcification on CT is helpful to differentiate CP type, it is not consistently demonstrated (as seen in our second case).

In our retrospective analysis of 5 cases, we found that each variant of CP had characteristic IOUS features. Particularly, IOUS can pick up small calcifications that are often missed on other imaging modalities. This can aid the surgeon in deciding the radicality of resection. Although promising, IOUS has a steep learning curve, and its correct interpretation is often subjective and explorer dependent [5]. Further studies with larger samples are needed to reaffirm the imaging correlation and its utility in intraoperative decision-making.

## Conclusion

Intraoperative sonography can help identify specific tumor characteristics and appearances differentiating papillary from adamantinomatous variant of CP. It can thus aid in intraoperative decision-making regarding the extent of resection.

## Data Availability

No datasets were generated or analysed during the current study.

## Abbreviations

IOUSG: Intraoperative Ultrasonography
CP: Craniopharyngioma
ACP: Adamantinomatous craniopharyngioma
PCP: Papillary craniopharyngioma
T1WMRI: T1 weighted magnetic resonance imaging
T2WMRI: T2 weighted magnetic resonance imaging
CT: Computed tomography

## References

[1] Adamson TE, Wiestler OD, Kleihues P, Yaşargil MG (1990) Correlation of clinical and pathological features in surgically treated craniopharyngiomas. J Neurosurg 73(1):12–17. 10.3171/jns.1990.73.1.0012 2352012 10.3171/jns.1990.73.1.0012

[2] Biswas C, Mansur G, Wu KC, Prevedello DM, Ghalib L (2024) Practical application of precision oncology in adult onset craniopharyngiomas. Front Endocrinol 15. 10.3389/fendo.2024.1488958 10.3389/fendo.2024.1488958 PMC11615394 39634188

[3] Brastianos PK et al (2014) Exome sequencing identifies BRAF mutations in papillary craniopharyngiomas. Nat Genet 46(2):161–165. 10.1038/ng.2868 24413733 10.1038/ng.2868 PMC3982316

[4] Brastianos PK et al (2023) BRAF–MEK inhibition in newly diagnosed papillary craniopharyngiomas. N Engl J Med 389(2):118–126. 10.1056/NEJMoa2213329 37437144 10.1056/NEJMoa2213329 PMC10464854

[5] Brugada-Bellsolà F, Rodríguez PT, González-Crespo A et al (2024) Intraoperative ultrasound and magnetic resonance comparative analysis in brain tumor surgery: a valuable tool to flatten ultrasound’s learning curve. Acta Neurochir 166:337. 10.1007/s00701-024-06228-2 39138764 10.1007/s00701-024-06228-2

[6] Finger G, Wu KC, Godil SS, Carrau RL, Hardesty D, Prevedello DM (2023) Ultrasound-guided endoscopic endonasal resection of sellar and suprasellar craniopharyngiomas. Front Surg 10:1073736. 10.3389/fsurg.2023.1073736 36896257 10.3389/fsurg.2023.1073736 PMC9990524

[7] Freda PU, Post KD (1999) Differential diagnosis of sellar masses. Endocrinol Metab Clin North Am 28(1):81–117. 10.1016/S0889-8529(05)70058-X 10207686 10.1016/s0889-8529(05)70058-x

[8] Hengartner AC, Prince E, Vijmasi T, Hankinson TC (2020) Adamantinomatous craniopharyngioma: moving toward targeted therapies. Neurosurg Focus FOC 48(1):E. 10.3171/2019.10.FOCUS19705 10.3171/2019.10.FOCUS19705 31896087

[9] Lee IH, Zan E, Bell WR, Burger PC, Sung H, Yousem DM (2016) Craniopharyngiomas: radiological differentiation of two types. J Korean Neurosurg Soc 59(5):466–470. 10.3340/jkns.2016.59.5.466 27651864 10.3340/jkns.2016.59.5.466 PMC5028606

[10] Louis DN et al (2021) The 2021 WHO classification of tumors of the central nervous system: a summary. Neuro-Oncol 23(8):1231–1251. 10.1093/neuonc/noab106 34185076 10.1093/neuonc/noab106 PMC8328013

[11] Marcus HJ, Vercauteren T, Ourselin S, Dorward NL (2017) Intraoperative ultrasound in patients undergoing transsphenoidal surgery for pituitary adenoma: systematic review. World Neurosurg 106:680–685. 10.1016/j.wneu.2017.07.054 28736351 10.1016/j.wneu.2017.07.054

[12] Moiyadi AV (2016) Intraoperative ultrasound technology in neuro-oncology practice—current role and future applications. World Neurosurg 93:81–93. 10.1016/j.wneu.2016.05.083 27268318 10.1016/j.wneu.2016.05.083

[13] Silveira-Bertazzo G, Manjila S, Carrau RL, Prevedello DM (2020) Expanded endoscopic endonasal approach for extending suprasellar and third ventricular lesions. Acta Neurochir (Wien) 162(10):2403–2408. 10.1007/s00701-020-04368-9 32385641 10.1007/s00701-020-04368-9

[14] Unsgaard G et al (2005) Ability of navigated 3D ultrasound to delineate gliomas and metastases – comparison of image interpretations with histopathology. Acta Neurochir (Wien) 147(12):1259–1269. 10.1007/s00701-005-0624-1 16172831 10.1007/s00701-005-0624-1

[15] Todeschini AB, Montaser AS, Shahein M, Revuelta JM, Otto BA, Carrau RL, Prevedello M (2018) Endoscopic endonasal approach to a suprasellar craniopharyngioma. J Neurol Surg Part B Skull Base 79:S237–S238. 10.1055/s-0038-1623526 10.1055/s-0038-1623526 PMC5868924 29588880

